# Identification and Comparative Analysis of the *Tegillarca granosa* Haemocytes MicroRNA Transcriptome in Response to Cd Using a Deep Sequencing Approach

**DOI:** 10.1371/journal.pone.0093619

**Published:** 2014-04-01

**Authors:** Yongbo Bao, Lili Zhang, Yinghui Dong, Zhihua Lin

**Affiliations:** Zhejiang Key Laboratory of Aquatic Germplasm Resources, Zhejiang Wanli University, Ningbo, Zhejiang, China; University of Nevada School of Medicine, United States of America

## Abstract

**Background:**

MicroRNAs (miRNAs) are endogenous non-coding small RNAs (sRNAs) that can base pair with their target mRNAs, which represses their translation or induces their degradation in various biological processes. To identify miRNAs regulated by heavy metal stress, we constructed two sRNA libraries for the blood clam *Tegillarca granosa*: one for organisms exposed to toxic levels of cadmium (Cd) and one for a control group.

**Results:**

Sequencing of the two libraries and subsequent analysis revealed 215 conserved and 39 new miRNAs. Most of the new miRNAs in *T. granosa* were up- or down-regulated in response to Cd exposure. There were significant differences in expression between the Cd and control groups for 16 miRNAs. Of these, five miRNAs were significantly up-regulated and 11 were significantly down-regulated in the Cd stress library. Potential targets were predicted for the 16 differential miRNAs in pre-miRNAs identified according to sequence homology. Some of the predicted miRNA targets are associated with regulation of the response to stress induced by heavy metals. Five differentially expressed miRNAs (Tgr-nmiR-8, Tgr-nmiR-21, Tgr-miR-2a, Tgr-miR-10a-5p, and Tgr-miR-184b) were validated by qRT-PCR.

**Conclusion:**

Our study is the first large-scale identification of miRNAs in *T. granosa* haemocytes. Our findings suggest that some miRNAs and their target genes and pathways may play critical roles in the responses of this species to environmental heavy metal stresses.

## Introduction

MicroRNAs (miRNAs) are a class of conserved, 20–25-nucleotide (nt), non-coding RNAs that depend on the RNAi machinery for maturation and function, and can mediate cleavage or translational repression of their target mRNAs by preferentially binding to the untranslated region of many protein-coding genes [1]. The breadth and importance of miRNA-directed gene regulation are of increasing interest as more miRNAs and their regulatory targets and functions are discovered. Recent functional studies have revealed that miRNAs control many other cellular functions, including proliferation, metabolism, apoptosis, and immunity [1], [2].

Pollution of marine ecosystems by heavy metals is a global environmental problem. The ecosystem of the east coast of China has been damaged as a result of industrial development [3], [4]. Heavy metals are among the most harmful elemental pollutants and are of particular concern because of their toxicity to humans. Cd ranks among the seven most common heavy metals (Cd, Cr, Cu, Hg, Ni, Pb, and Zn) released into the environment [5], and accumulates in a great number of marine invertebrates, especially bivalve and gastropod molluscs. Cd is the most serious heavy-metal pollutant in the East China Sea, especially along the Zhejiang coast, due to emissions from electroplating, chemical, and electrical plants. Thus, we selected Cd as a stimulant for challenge experiments.

Recently, more and more studies indicate that miRNAs are involved in response to heavy metals, especially in plant [6]. Several new putative small RNAs from rice and rape in response to Cd were identified [7], [8]. 12 miRNAs and their targets reveals their differential regulation by Hg exposure were identified in medicago *Medicago truncatula* [9]. Transcriptional and post-transcriptional gene regulation is important for the response to metal exposure or metal deficiency. However, the regulatory network in metal homeostasis is largely unknown in animals, especially in mollusk. Analysis of miRNAs and their targets involved in heavy metal stress, mediation may provide a new insight into understanding of mollusk stress response mechanisms.

Marine clams are an excellent model system for studies on resistance to natural multi-stresses, since their intertidal environment involves exposure to anoxic stress, heavy metals, and pollutants. Filter-feeding lamellibranch molluscs, such as clams, have a remarkable ability to accumulate high tissue levels of trace metals, since these animals have mechanisms for detoxification of heavy metals [10], [11]. Biological responses to cadmium are complex, and are far from being fully understood.

In the present study we used the blood clam *Tegillarca granosa* as a model to assess the involvement of miRNAs in organismal responses to Cd stress. We constructed small-RNA (sRNA) libraries from haemocytes of *T. granosa* and sequenced the libraries using Illumina sRNA deep-sequencing technology. The sequencing data were analysed to identify conserved and novel miRNAs and their targets. This work represents the first report of miRNAs identified in a marine clam, may be helpful for a better understanding of the mechanisms of Cd accumulation and tolerance in mollusk.

## Materials and Methods

### Ethics Statement

No specific permits were required for the described field study. No specific permissions were required for this location and activities. The location is not privately-owned or protected in anyway and the field studies did not involve endangered or protected species.

### Challenge and sample collection

Blood clams, averaging about 30 mm in shell length, were collected from a clam farm in Ningbo and acclimatized in seawater tanks (10 m^3^) for one week before processing. The seawater temperature was 25±1.0°C and the salinity was 30‰ throughout the experiments. For heavy metals challenge experiment, blood clam were divided in to four tanks and were exposed to heavy metals of Cd^2+^ with the final gradient concentration of 25, 250 and 500 μg/L. The fourth tank was served as control group. The seawater was changed daily and metal stock solution was added to the seawater every day. After 24 h exposure, the haemocytes from each group were collected from the control and the treated groups for RNA extraction and cDNA synthesis. Ten blood clams from control group and 250 μg/L challenge group were randomly sampled respectively for small RNAs library construction.

### Small RNAs library construction and deep sequencing

Two small RNA libraries pooled from control group (C) and heavy metal treatment group (E) were constructed. Total RNA was extracted using Trizol according to the manufacturer's protocol, and the quantity of RNA was examined by using an Agilent 2100 Bioanalyzer. After collecting RNA with small size ranging from 20 nt to 30 nt, a pair of Illumina proprietary adaptors were ligated to their 5′ and 3′ ends, followed by reverse transcription. The two generated small cDNA libraries were amplified by PCR with primers complementary to the adaptor sequences. Subsequently, the libraries were deep sequenced by Illumina Hiseq2000 according to the manufacturer's instructions.

### Bioinformatic analysis of sequencing data

Millions of short reads from next generation miRNA sequencing would be processed by several steps. An initial filtering step was performed to exclude reads of low quality as well as reads that contain too many missing nucleotides based on the quality scores. Then, these raw sequencing data were filtered by eliminating adaptor contaminants to generate usable reads with size ≥15 nt. Thirdly, the clean reads were aligned to Rfam and GenBank and rRNA, tRNA, snRNA, scRNA and snoRNA were discarded from the small RNA sequences. The obtained reads were also aligned against the miRBase19.0 (http://mirbase.org/) and reference genome for conserved and novel miRNA identification combined with stem-loop structure prediction. Raw sequencing data for the transcriptome have been deposited in the NCBI Sequence Read Archive with an accession number, SRR1146538.

### Analysis of conserved and novel miRNAs

To identify the conserved miRNAs in blood clam, small RNAs deep-sequencing data were aligned with miRBase19.0, Rfam, Repeat, EST databases to search for known miRNAs with complete matches. Reads that did not match databases were marked as unannotated. To predict novel miRNAs, the unmatched data sets were aligned with *Crassostrea gigas* genomic sequence (http://www.ncbi.nlm.nih.gov/ genome/?term = Crassostrea%20gigas). To analyze whether the matched sequence could form a suitable hairpin (the secondary structure of the small RNA precursor), sequences surrounding the matched sequence were extracted. The second structure was predicted by miRDeep2.0 [12].

### Differential expression analysis of miRNAs

To compare miRNAs expression data between C and E library, miRNAs expression in each library was normalized to obtain the expression of transcripts per million using mapped (FPKM) method. The abundance of each data set was normalized to 10 million. The fold-change and *P*-value were calculated from the normalized expression. When |log2Ratio| ≥1and *P*-value ≤0.05, it was be seen as differential expression.

### Quantitative real-time PCR of miRNAs

To validate and characterize the differentially expressed miRNAs identified using high-throughput sequencing, five miRNAs (Tgr-nmiR-8, Tgr-nmiR-21, Tgr-miR-2a, Tgr-miR-10a-5p and Tgr-miR-184b) were selected, and we analyzed their relative expression levels in haemocytes at challenge with the gradient concentration of 25, 250 and 500 μg/L Cd^2+^. NCode miRNA First-Strand cDNA Synthesis and qRT-PCR Kits (Ambion, USA) was used for polyadenylation and reverse transcription of miRNAs for use in two-step quantitative RT-PCR. The cDNA was then used for real time PCR on a ABI Fast7500 instrument system (ABI, USA) using SYBR green-based real time PCR with miRNA-specific forward primer and universal reverse primer (Table 1). U6 was used as the internal control. In a 96-well plate, each sample was run in triplicate along with the internal control gene. The each miRNA expression level was presented as 2^−ΔΔCt^ means ± SE (n = 3), and error bars indicate the standard error of 2^−ΔΔCt^ mean values. The data were then subjected to Student t-test to determine difference in the mean values among the treatment. The data were then subjected to one-way analysis of variance (one-way ANOVA) followed by an unpaired. A *P*-value <0.05 was considered to be significant. Statistical analysis was performed using software SPSS 19.

**Table 1:** Sequences of primers used in this study for qRT-PCR.

Primers	sequence (5′-3′)
Tgr-nmiR-8	AATGGCACTGGTAGAATTCACGG
Tgr-nmiR-21	TACCCTGTAGATCCGAATTTGT
Tgr-miR-2a	TCACAGCCAGCTTTGATGAGCA
Tgr-miR-10a-5p	TACCCTGTAGATCCGAATTTGTG
Tgr-miR-184b	TGGACGGAGAACTGATAGGGC
U6	ATTGGAACGATACAGAGAAGATTAG

### Validated differential miRNA Target gene Prediction

Finally, to get an idea of the potential role of differentially expressed miRNAs and of their general function, we performed a three-phase method (miRanda) for target site identification by searching 454 transcriptome sequencing library of *T. granosa* [13]. The three phases are as follows: sequence-matching to assess first whether two sequences are complementary and possibly bind; free energy calculation (thermodynamics) to estimate the energetics of this physical interaction; and evolutionary conservation as an informational filter. To identify miRNA target functions and classifications, as well as the metabolic regulatory networks associated with blood clam miRNAs and their targets, we conducted GO analysis by running a BLASTX search for each target sequence against UniProt database. The best hits were used to validate the target gene functions and metabolic pathways regulated by miRNAs. The molecular functions of the gene products and the subcellular locations where these products are located were obtained from UniProt-GO Annotation database.

## Results and Discussion

### Sequence analysis of sRNAs

**Figure 1:**
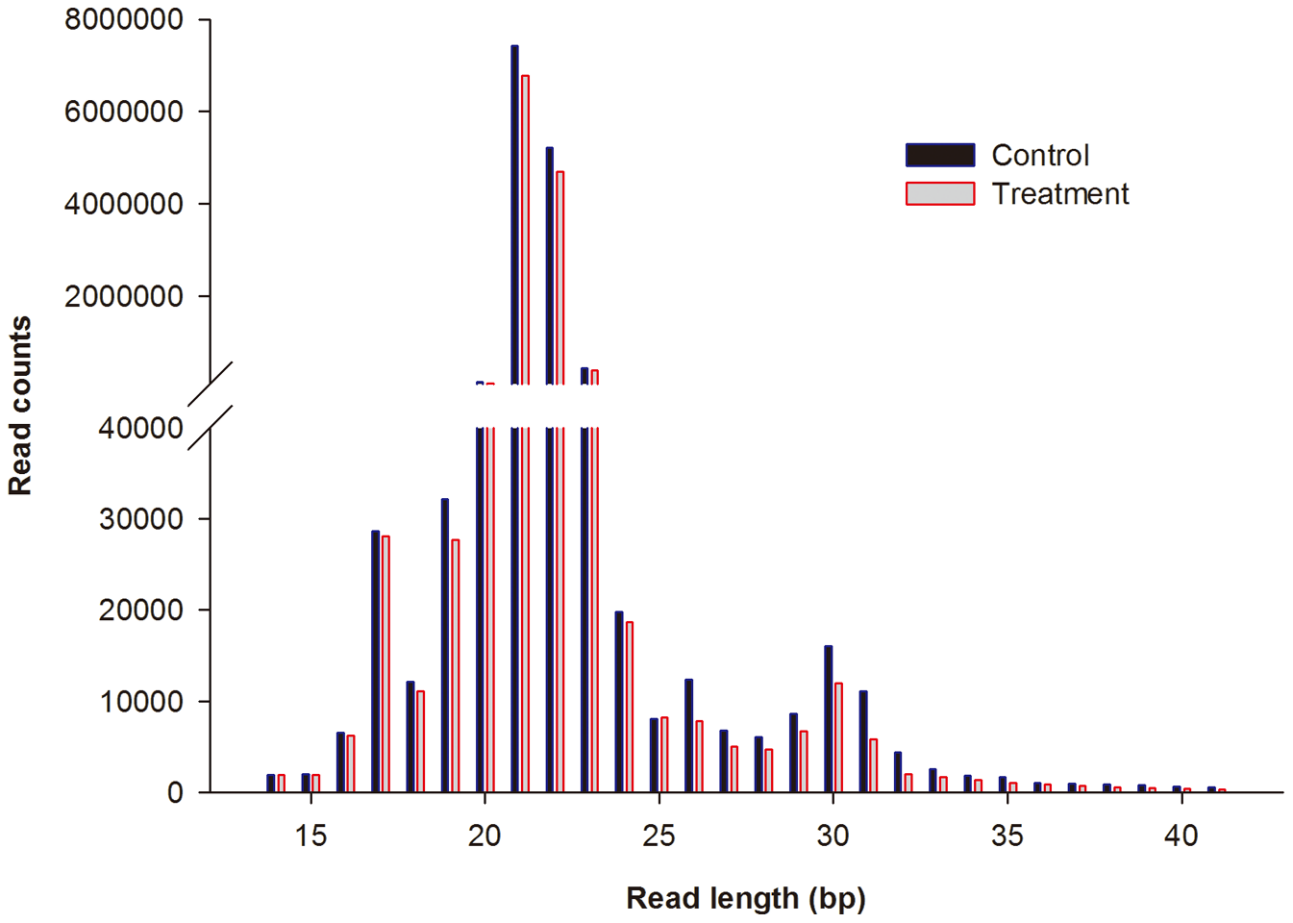
Length distribution and abundance of sequenced small RNAs from *Tegillarca granosa*.

To identify miRNAs expressed in response to Cd, sRNA libraries were generated for *T. granosa* control (C) and exposure (E) groups. The libraries were sequenced using Illumina sRNA deep-sequencing technology. In total, 13,408,818 and 12,138,445 clean reads were obtained from the C and E libraries, respectively (Table 2). The sRNA size distribution was similar for the two libraries, and the majority of the sRNAs were 21–23 nt in size (Figure 1). The most abundant size class was 21 nt, which accounted for 55.32% and 55.81% of the total reads in the C library and E library, respectively, followed by 22 nt (38.92% and 38.69%) and 23 nt (3.28% and 3.29%). These results are consistent with the miRNA size in the cherry *Eugenia uniflora* [14] and sea cucumber *Apostichopus japonicus* [15], Pearl oyster *Pinctada martensii* [16]. Other studies showed that 22 nt is the dominant read length for insects such as *Blattella germanica* [17], *Locusta migratoria* [18] and *Aedes albopictus* [19], In plant such as *Citrus trifoliate* [20], *Cucumis sativus* [21] and *Vitis vinifera*, 24 nt was the dominant miRNA length [22].

**Table 2:** Summary statistics of small RNA sequencing in *T. granosa*.

	C library		E library	
	**Clean reads**	**Unique Reads**	**Clean reads**	**Unique Reads**
	(13408818)	(51726)	(12138445)	(43505)
**Referred genome**	421521	5413	389889	5414
**Rfam alignment**	411586	5456	380266	5671
**Repeat alignment**	9298	2797	9041	2767
**EST alignment**	9915	3594	9331	3547
**miRNA mature**	394890	1728	362070	1730
**Conserved miRNA**	215		243	
**Novel miRNA**	38		41	

### Identification of conserved miRNAs

**Figure 2:**
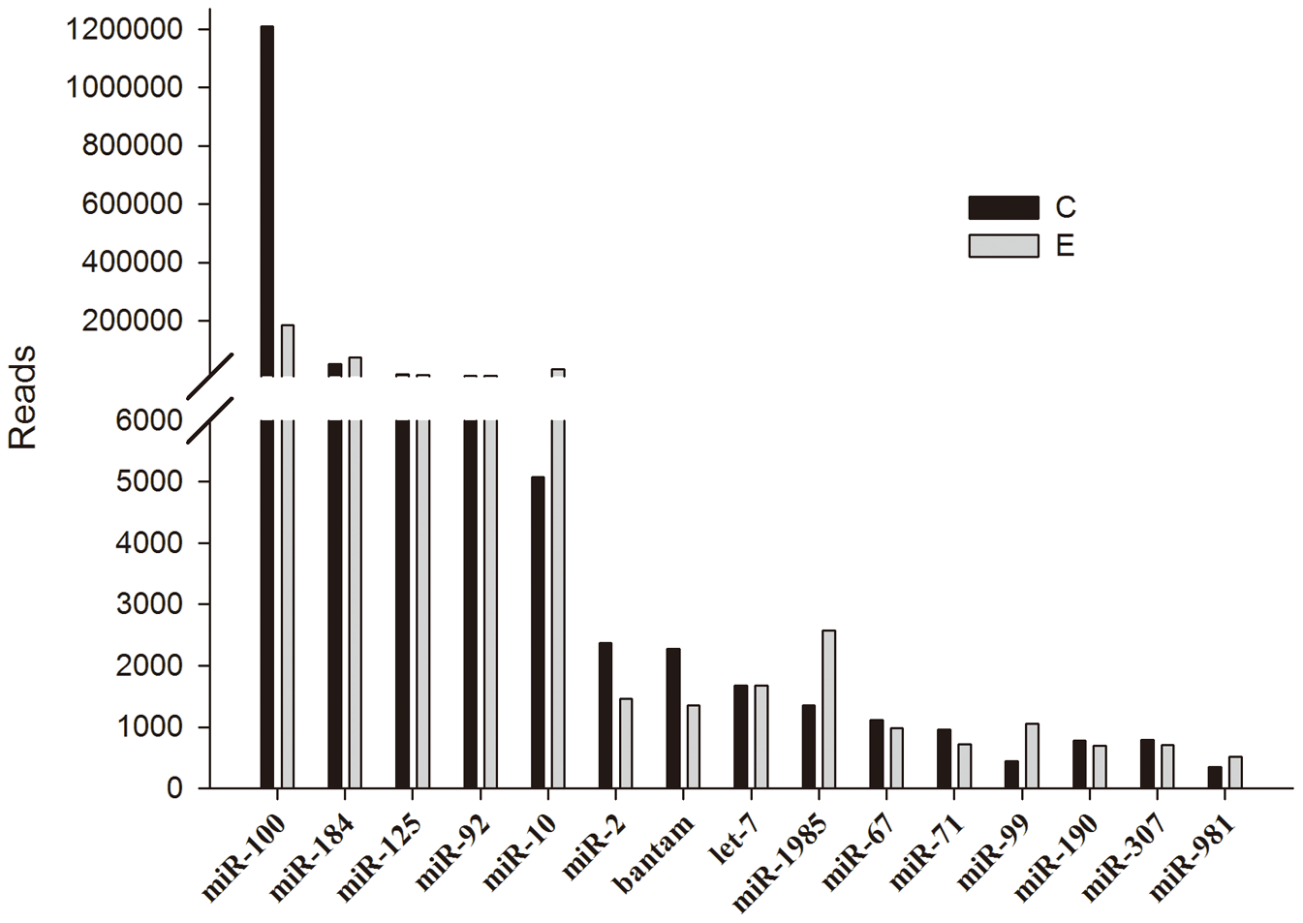
Number of identified miRNAs in each conserved miRNA family in *T. granosa*.

Using the genome of the Pacific oyster *Crassostrea gigas* as a reference, 215 conserved miRNAs were identified in the C library and 243 in the E library (Table 2; Table S1). Figure 2 shows the 15 most abundant conserved miRNAs with more than 300 reads. The most abundant was miR-100, with 1,210,410 reads in the C library and 184,304 reads in the E library, followed by miR-184, miR-125, and miR-92. Among these, miR92 and miR-184 were also identified as one of the most abundant miRNAs in *A. japonicas* [15], *P. martensii* [16] and *C. quinquefasciatus* [19], indicating that these miRNAs are highly expressed and function as negative regulators of gene expression in various organisms. miR100 targets mTOR, which is a serine/threonine protein kinase that regulates cell growth, proliferation, motility, and survival, protein synthesis, and transcription [23], [24]. The link between miR100 and mTOR signalling has been proved in many studies about human cancer researches [24], [25]. miR-184 is a single-copy gene that is evolutionarily conserved at the nucleotide level among diverse species [26]. Several targets for miR-184 have been described, including mediators of neurological development and apoptosis, and it has been suggested that miR-184 plays an essential role in development [27]. miR-125, which is also a highly conserved miRNA among diverse species, plays crucial roles in many different cellular processes such as differentiation, proliferation, and apoptosis by targeting many different transcription factors such as matrix metalloproteases and growth factors [28]. Two miRNAs with specific names, bantam and lethal-7 (let-7), were highly expressed in both libraries. Bantam is essential target of the Hippo signalling pathway and regulates cell proliferation, death, and survival [29]. The let-7 gene, first discovered as a key developmental regulator, was one of the first two known miRNAs [30]. Let-7 has been implicated in post-transcriptional control of innate immune responses to pathogenic agents [31]. In our study, let-7 was abundant in both libraries with almost equal numbers of reads, which indicates that is not differentially expressed in response to Cd stress.

### Identification of novel miRNA candidate

**Figure 3:**
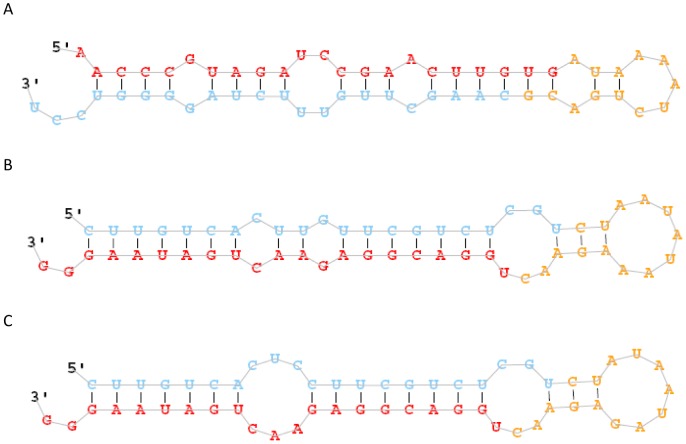
Predicted stem-loop secondary structures of *T. granosa* coding candidate miRNAs. Folding and free energy calculations were determined with miRDeep2. Dominant forms of the mature miRNAs are indicated in red color (A: Tgr-nmiR-13, B: Tgr-nmiR-32; C:Tgr-nmiR-36).

We used the miRNA prediction software miRDeep, which predicts characteristic hairpin structures of pre-miRNAs and Dicer digestion sites, to predict novel miRNAs in the two libraries [12]. A total of 39 precursors of the unannotated miRNAs could form a proper secondary hairpin and were considered as novel miRNAs. These included 39 miRNAs from the C library and 41 miRNAs from the E library, of which 39 miRNAs were common to both libraries (Table S2). The following three criteria were used to evaluate reads with potential miRNA-like hairpins: (1) total conservation of the first 15 nt, (2) the free energy for the hairpin, and (3) the flanking sequences for the hairpin. This analysis identified the 39 novel miRNA candidates listed in Table 3. Figure 3 shows the secondary structure for the three most abundant candidates (Tgr-nmiR-13, Tgr-nmiR-32, and Tgr-nmiR-36) predicted using RNA-fold software (http://rna.tbi.univie.ac.at/cgi-bin/RNAfold.cgi). These candidates had a concentrated length distribution of 18–23 nt. Novel miRNA candidates should be further validated by direct cloning to overcome the inability of bioinformatic analysis to precisely predict the position of the mature miRNA within the stem–loop structure.

**Table 3:** Novel miRNA candidate identified from blood clam.

miRNA	provisional id	mature read count	consensus mature sequence	miRDeep2 score
Tgr-nmiR-1	gi|431998402| gb|KB200869.1|_77	1225	ugagguaguagguuguauaguu	626.5
Tgr-nmiR-2	gi|431997034|gb|KB202237.1|_190	951	cgggacuacgucaacuacuagc	486.8
Tgr-nmiR-3	gi|431997682|gb|KB201589.1|_137	83	ugagauucaacuccuccaacugc	44.1
Tgr-nmiR-4	gi|431997812|gb|KB201459.1|_126	54	uaaucucagcugguaauucuga	28.7
Tgr-nmiR-5	gi|431999620|gb|KB199651.1|_9	21	ugaacacagcuggugguaucuucu	2.4
Tgr-nmiR-6	gi|431997967|gb|KB201304.1|_112	1408	uaucacagccugcuuggaucagca	2.3
Tgr-nmiR-7	gi|431996252|gb|KB203019.1|_245	14	aggcaagauguuggcauagcuga	2.1
Tgr-nmiR-8	gi|431995970|gb|KB203301.1|_262	225	aauggcacugguagaauucacgg	2
Tgr-nmiR-9	gi|431997967|gb|KB201304.1|_103	3150	uaucacagccugcuuggaucagu	1.9
Tgr-nmiR-10	gi|431998402|gb|KB200869.1|_75	122	ucccugagaccauaauuu	1.9
Tgr-nmiR-11	gi|431996988|gb|KB202283.1|_199	38	ugagacaguguguccucccu	1.9
Tgr-nmiR-12	gi|431997967|gb|KB201304.1|_105	2322	uaucacagccagcuuugaugagu	1.8
Tgr-nmiR-13	gi|431998402|gb|KB200869.1|_79	16465	aacccguagauccgaacuugug	1.7
Tgr-nmiR-14	gi|431996727|gb|KB202544.1|_226	91	uauugcacuugucccggccuuuc	1.7
Tgr-nmiR-15	gi|431995997|gb|KB203274.1|_249	17	ugucauggaguugcucucuuua	1.7
Tgr-nmiR-16	gi|431997812|gb|KB201459.1|_128	17	ugaguauuacaucagguacuga	1.7
Tgr-nmiR-17	gi|431997967|gb|KB201304.1|_110	775	uaucacagccugcuuggaucag	1.7
Tgr-nmiR-18	gi|431997966|gb|KB201305.1|_115	197	agcugccugaugaagagcuguac	1.6
Tgr-nmiR-19	gi|431995997|gb|KB203274.1|_258	17	ugucauggaguugcucucuuua	1.6
Tgr-nmiR-20	gi|431998401|gb|KB200870.1|_81	61	gugagcaaaguuucagguguau	1.6
Tgr-nmiR-21	gi|431999142|gb|KB200129.1|_31	3280	uacccuguagauccgaauuugu	1.6
Tgr-nmiR-22	gi|431995733|gb|KB203538.1|_283	13	uuuugauuguugcucagaaagcc	1.5
Tgr-nmiR-23	gi|431997966|gb|KB201305.1|_117	131	gagcugccaaaugaagggcugu	1.5
Tgr-nmiR-24	gi|431997340|gb|KB201931.1|_164	5295	cuuggcacuggcggaauaaucac	1.5
Tgr-nmiR-25	gi|431996727|gb|KB202544.1|_224	8797	aauugcacuugucccggccugc	1.4
Tgr-nmiR-26	gi|431998569|gb|KB200702.1|_63	1085	uuugugaccguuauaaugggca	1.4
Tgr-nmiR-27	gi|431998569|gb|KB200702.1|_61	49	cuaaguacuggugccgcgggag	1.4
Tgr-nmiR-28	gi|431996988|gb|KB202283.1|_191	124	uaauacugucagguaaagaugucc	1.3
Tgr-nmiR-29	gi|431997340|gb|KB201931.1|_167	208	uacuggccugcaaaaucccaac	1.3
Tgr-nmiR-30	gi|431999319|gb|KB199952.1|_24	1853	auuuggcacuuguggaauaaucg	1.1
Tgr-nmiR-31	gi|431996988|gb|KB202283.1|_196	6209	ugccauuuuuaucagucacugug	1.1
Tgr-nmiR-32	gi|431998924|gb|KB200347.1|_44	16758	uggacggagaacugauaaggg	1.1
Tgr-nmiR-33	gi|431996988|gb|KB202283.1|_205	24	gacagauguauccaucugag	1
Tgr-nmiR-34	gi|431996727|gb|KB202544.1|_222	10851	aauugcacuugucccggccugc	0.9
Tgr-nmiR-35	gi|431997967|gb|KB201304.1|_113	9	ucagcugucaugaugccuuccu	0.8
Tgr-nmiR-36	gi|431998924|gb|KB200347.1|_42	16758	uggacggagaacugauaaggg	0.7
Tgr-nmiR-37	gi|431997682|gb|KB201589.1|_135	111	ucagaucuaacucuuccagcuca	0.7
Tgr-nmiR-38	gi|431997600|gb|KB201671.1|_150	9	ucagcaguuguaccacugauuug	0.4
Tgr-nmiR-39	gi|431995996|gb|KB203275.1|_260	8	ugacuagauccacacucaucca	0

### miRNAs differentially expressed in two groups

The main purpose of the study was to identify miRNAs involved in Cd stress and resistance. The threshold we used to screen for miRNA up- or down-regulation was a two-fold change between the groups for a *P* value <0.05. According to changes in the relative miRNA abundance between the two libraries, significant differences in expression between the control and Cd groups were observed for16 miRNAs (Table 4). In comparison to the control group, five miRNAs were significantly up-regulated, and 11 miRNAs were significantly down-regulated in the Cd group. The difference in expression for the majority of these miRNAs ranged from two- to approximately six-fold. Among the down-regulated miRNAs, the novel miRNA Tgr-nmiR-21 had the highest fold-change (6.174), followed by Tgr-nmiR-8 and Tgr-miR-2a, for which the change was greater than six-fold. Among the up-regulated miRNAs, Tgr-miR-33-5p had the highest fold-change (infinite), followed by Tgr-miR-10a-5p and Tgr-miR-184b, for which the change was greater than five-fold. The miR-2 family is an invertebrate-specific family of miRNAs that are probably involved in neural development and maintenance [32]. Tgr-miR-33-5p is possibly involved in cellular and developmental processes according to previous studies in other species [33], [34]. The presence of miR-10 has been detected in a diverse range of bilaterian animals, and it is one of the most widely distributed miRNAs in animals. A number of Hox genes are regulated by miR-10. These genes encode transcription factors that are important in embryonic development [35]. Tgr-nmiR-21 targets ring finger proteins, which are mediators of ubiquitin ligase activity. The ubiquitination system functions in a wide variety of cellular processes, including immune responses, inflammation, and responses to stress and extracellular modulators [36], [37]. Identification of Tgr-nmiR-21 and its target ring finger proteins in relation to ubiquitin will help to further our understanding of the regulatory mechanism for cell wall modification under heavy metal stress.

**Table 4:** Sixteen differentially expressed miRNAs regulated greater than 2-fold in control and Cd challenge library.

MiR-name	Fold-change	P-value	regulated	Sig-lable
Tgr-nmiR-21	6.1735	7.13E-07	down	**
Tgr-nmiR-8	6.0462	6.98E-04	down	**
Tgr-miR-2a	6.0061	0.026	down	*
Tgr-miR-184	4.8865	3.52E-05	down	**
Tgr-miR-2001	4.6893	0.017	down	*
Tgr-nmiR-36	4.3933	1.68E-04	down	*
Tgr-miR-67	4.2249	9.27E-04	down	**
Tgr-miR-71-5p	4.2227	0.012	down	*
Tgr-miR-10	3.9791	4.54E-04	down	**
Tgr-nmiR-31	3.7909	9.38E-04	down	**
Tgr-mir-2	2.3860	0.031	down	*
Tgr-miR-71	3.0262	0.035	up	*
Tgr-miR-2a	3.2198	0.012	up	*
Tgr-miR-10a-5p	5.0112	4.44E-05	up	**
Tgr-miR-184b	5.3378	1.73E-04	up	**
Tgr-miR-33-5p	Inf	0.045	up	*

* represents fold change (log2)>1 or fold change(log2)<-1, and *P*-value<0.05, **represents P-value<0.01.

### qRT-PCR validation of differentially expressed miRNAs

**Figure 4:**
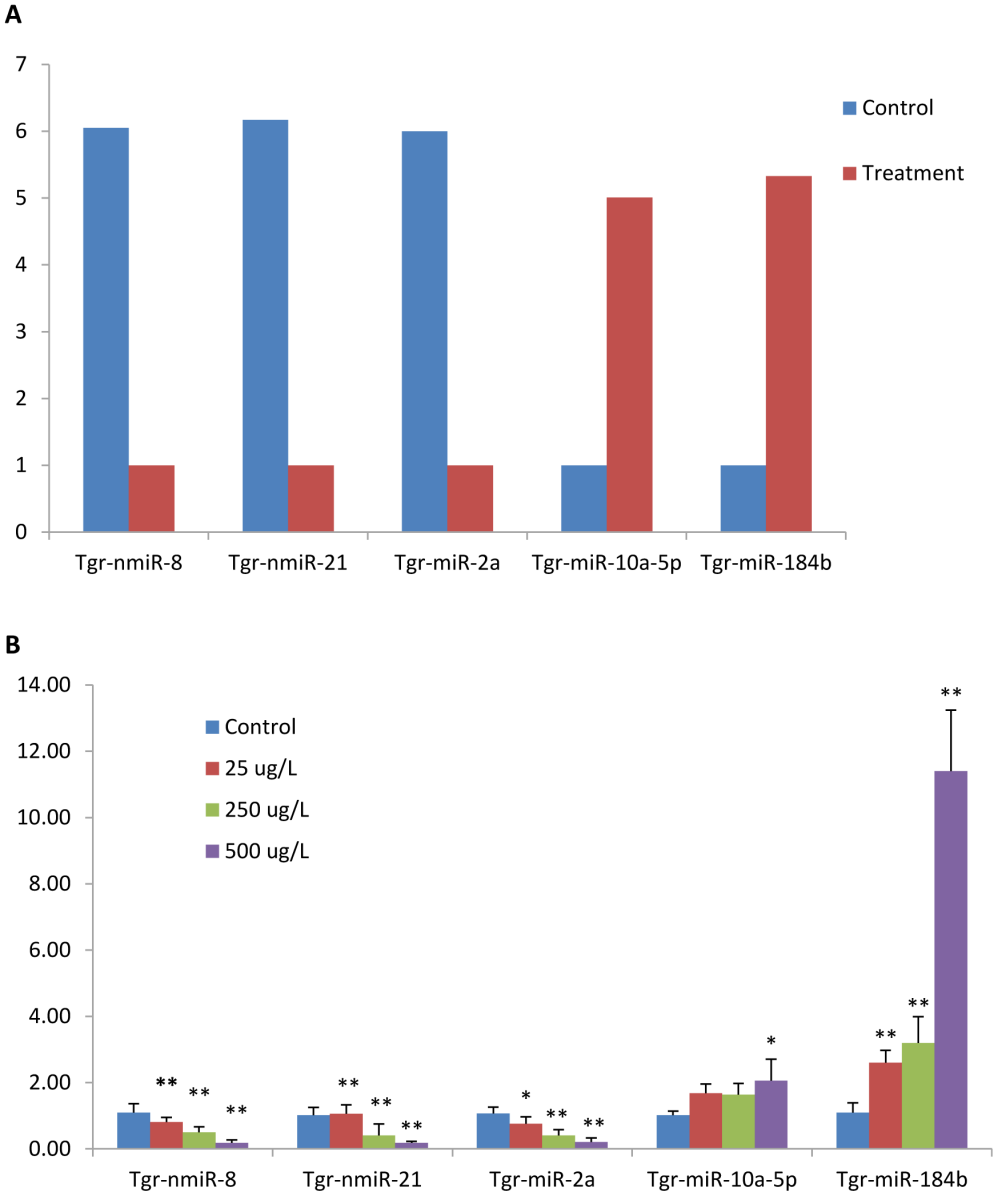
qRT-PCR validation of five differentially expressed miRNAs identified using Illumina small RNA deep sequencing. A. Fold change of five miRNAs that were differentially expressed between C and E library based on deep sequencing data. B. The relative expression abundance of the five miRNAs in haemocytes at challenge with the gradient concentration of 25, 250 and 500 μg/L Cd^2+^ by qRT-PCR. * *P*<0.05, ** *P*<0.01. The amount relative to the internal control U6 is expressed as mean ± SD (N = 3).

To validate the Illumina results, levels of randomly selected miRNAs (Tgr-nmiR-8, Tgr-nmiR-21, Tgr-miR-2a, Tgr-miR-10a-5p, and Tgr-miR-184b) were quantified by qRT-PCR in haemocytes on challenge with concentration gradients up to 25,250 and 500 μg/L Cd^2+^. The qRT-PCR results for miRNAs and mRNAs presented in Figure 4 demonstrate very good correspondence between the two platforms, except for Tgr-miR-10a-5p, which did not significantly decrease for 25, 250 μg/L Cd^2+^. The expression levels of these five miRNAs showed a significant dose–response relationship.

### Predicted miRNA targets and functional annotation

A total of 797 target genes were identified for the known miRNAs (Table S3). Among these genes, several genes were demonstrated involved in Cd challenge. Understanding of heavy metal-responsive gene expression and regulation is the first step to dissect the genetic and molecular basis of metal hyperaccumulation. The cation transport and diffusion facilitators (CDF) related protein which are targeted by Tgr-nmiR-21, CDF proteins are a recently discovered family of cation efflux transporters that might play an essential role in metal homeostasis and tolerance [38], heavy metal-transporting protein such as metal-transporting ATPase, disintegrin and metalloproteinase, glutamine synthetase and GTPase-activating-like protein, which are targeted by significant differentially miRNA: Tgr-nmiR-8, Tgr-miR-10, Tgr-miR-67. These target genes were also found in plant response to Cd stress [39]. Moreover, the main responses observed in blood clam heamocytes was the induction of genes involved in sulfur acquisition and assimilation. Many studies showed that sulphur-containing metabolites are much related to heavy metal homeostasis and detoxification [40]–[42].

**Figure 5:**
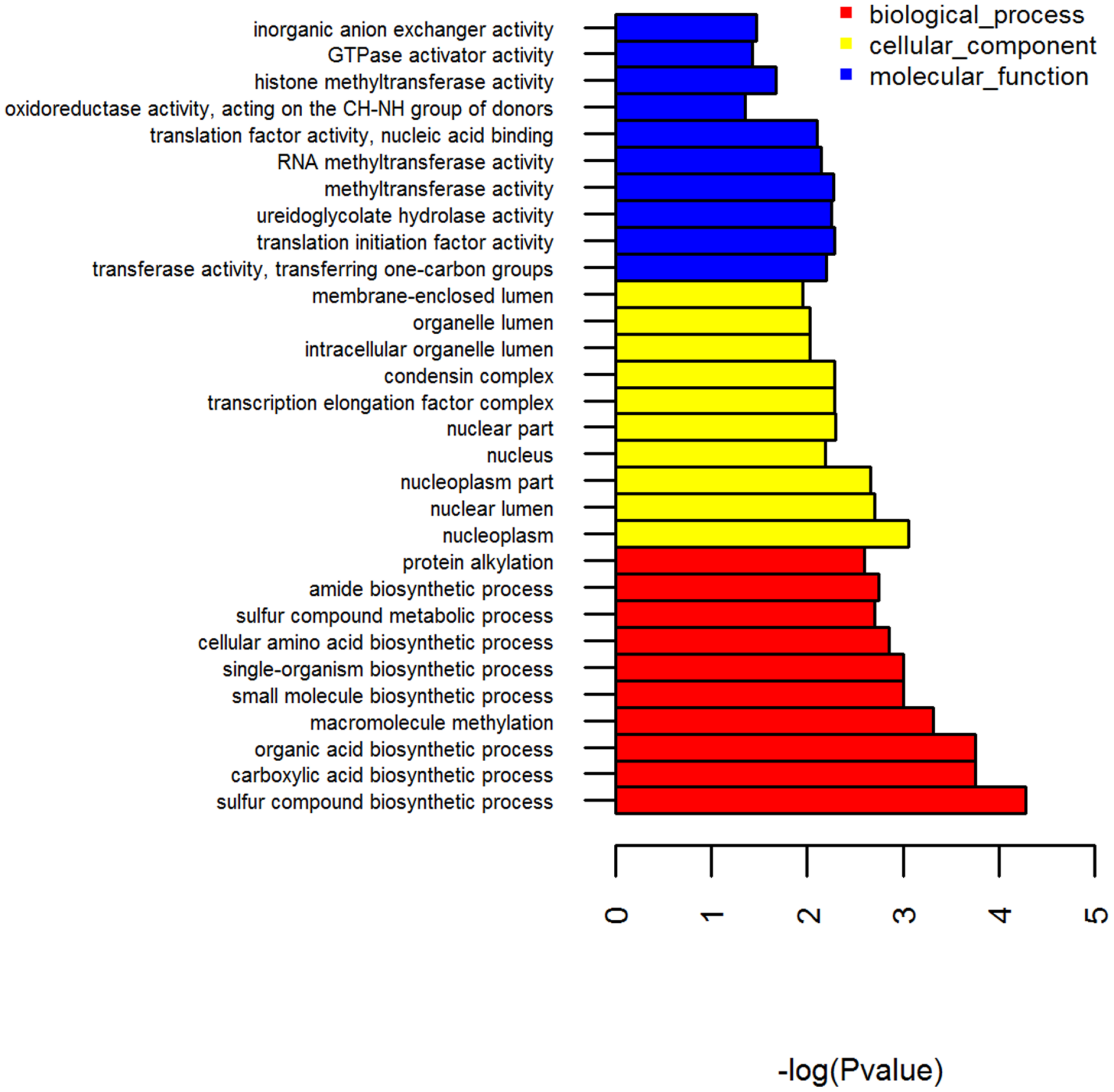
Gene categories and the distribution of target genes of the differentially expressed miRNAs in the conserved and novel pre-miRNA identified in *T. granosa*. The figure shows partial GO enrichment for the predicted target genes in molecular function, cellular component and biological processes.

**Figure 6:**
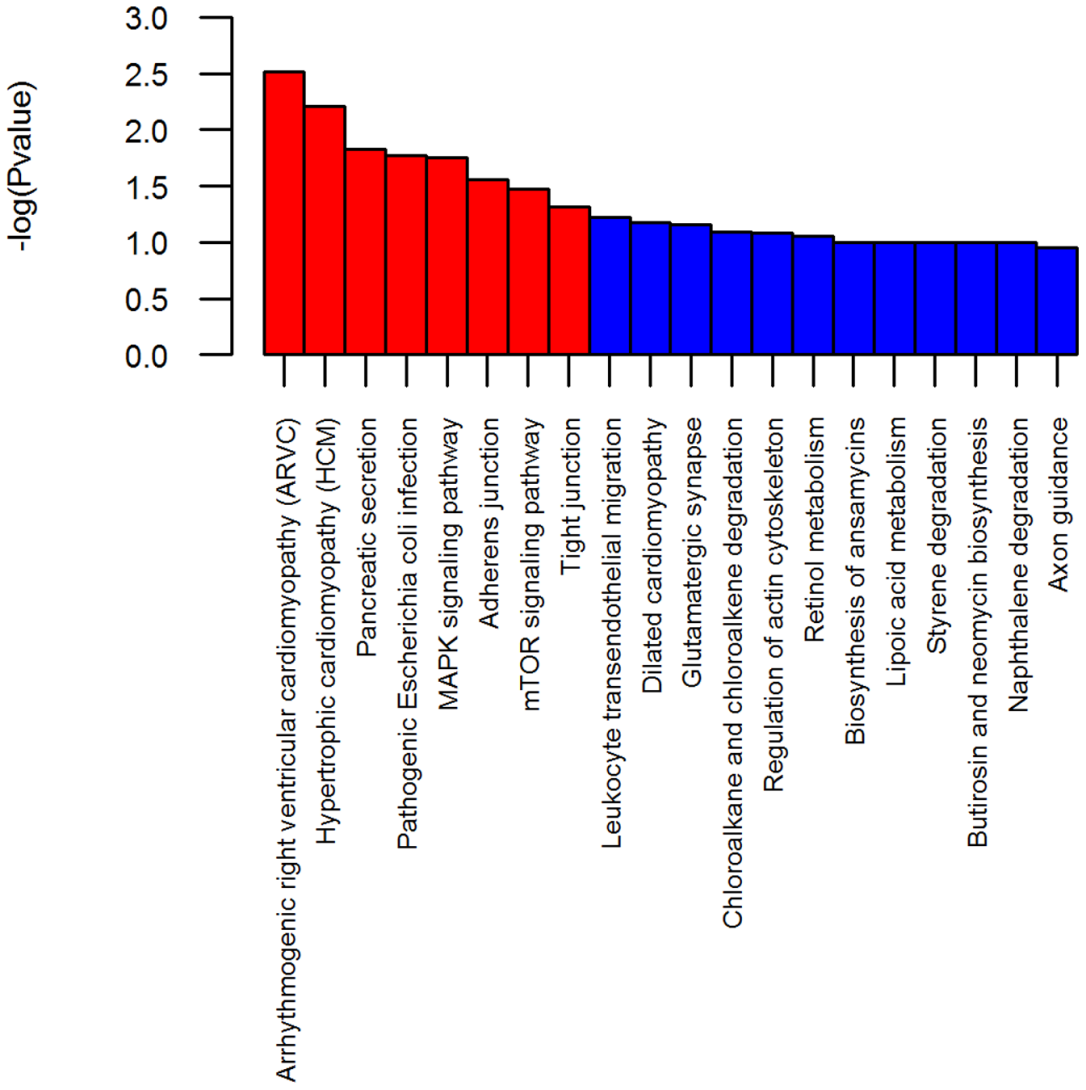
The most enriched KEGG pathways of target genes for differentially expressed miRNAs.

To describe the network of miRNAs and target genes involved in Cd stress and resistance, we constructed a regulatory network diagram. To gain insights into the biological implications of differentially expressed miRNAs, all targets regulated by the differentially expressed miRNAs identified were subjected to gene ontology (GO) analysis to evaluate their potential functions. GO categories include biological processes, cellular components, and molecular functions, as summarized in Figure 5. Sulfur compound biosynthetic processes, the nucleoplasm, and methyltransferase activity were the most significantly enriched for each of the categories. To identify the biological pathways affected by Cd stress and resistance, we performed KEGG pathway enrichment analysis for the differentially expressed genes. This analysis revealed 214 pathways enriched with miRNA targets (Table S4). Arrhythmogenic right ventricular cardiomyopathy (ARVC), hypertrophic cardiomyopathy, pancreatic secretion, pathogenic Escherichia coli infection, MAPK signalling, adherens junction, and tight junction pathways were among the most enriched pathways (Figure 6). The MAPK signalling pathway plays a significant role in responses to acute thermal stress and various heavy metals, as well as its possible involvement in either anti-apoptotic or pro-apoptotic events [11]. Sulfur compound biosynthetic and metabolic processes are Cd-sensitive and provide evidence of the importance of sulfide ions in metal tolerance in plants [43], [44]. Our results indicate that these two pathways are also involved in Cd detoxification in *T. granosa*. Previous studies in other animals revealed that the pancreatic secretion pathway can be induced by Cd and other heavy metals [45], [46]. Research has shown that Cd is initially an effective inhibitor of DNA methyltransferase (MeTase) activity and induces DNA hypomethylation; prolonged exposure results in DNA hypermethylation and enhanced DNA MeTase activity [47]. Overall, our GO and KEGG analysis results reveal miRNAs related to Cd or heavy metal metabolism and detoxification. Further study will focus on experimental validation of miRNAs of interest and their target genes and pathways.

## Conclusion

The aims of this study were to identify new miRNAs in *T. granosa* haemocytes and determine which miRNAs might be regulated by Cd stress. Our results have important implications for our understanding of gene regulation under Cd stress and make a significant contribution to the long-term goal of a complete miRNA profile for *T. granosa*. Further investigation of the functions of these miRNAs should increase our understanding of the roles of miRNAs in regulating Cd challenge and stress.

## Supporting Information

Table S1 **Identified conserved and novel miRNAs in** ***T. granosa*** **.**
(XLS)

Table S2 **Novel miRNAs predicted by miRDeep2 in** ***T. granosa*** **.**
(XLS)

Table S3 **Predicted differential miRNA target genes and functional annotation.**
(XLS)

Table S4 **KEGG pathway enrichment analysis for the differentially expressed genes.**
(XLS)
